# Reply to Letter to the Editor on “Not all biases are bad: equitable and inequitable biases in machine learning and radiology”

**DOI:** 10.1186/s13244-021-01088-1

**Published:** 2021-11-03

**Authors:** Mirjam Pot, Barbara Prainsack

**Affiliations:** 1 Department of Political Science, University of Vienna, Vienna, Austria; 2 Department of Global Health and Social Medicine, King’s College London, London, UK

**Keywords:** Artificial intelligence, Bias, Inequity

## Introduction

Iannessi et al. [1] commented on our paper “Not all biases are bad: equitable and inequitable biases in machine learning and radiology” [2]. We thank the authors for their critique. At the same time, we would like to take this opportunity to correct some misunderstandings.

In societies characterised by strong social inequities, these inequities are also inscribed in medicine, medical data, and data technologies. This means that the datasets and algorithms used for machine learning (ML) are never straightforward representations of people, bodies, and populations [3], but they are “funhouse mirrors” [4] that reflect how access to resources are distributed in a population. As a result—and this is what we argue in the paper—concerns about equity should ideally come in already at the stage of deciding whether or not a dataset is biased: The question of *what constitutes a bias* should be the starting point of the reflection. Is a dataset biased if it does not adequately represent the patient population of a particular hospital? A particular city or region? Or a country? What is a good criterion to decide whether a dataset is biased if we take into consideration equity considerations as well? Furthermore, even if biases cannot be avoided researchers and practitioners should reflect the possible effects of biases in particular with regard to equity. Is a specific bias likely to create inequitable health outcomes for particular patient groups? In other words, we suggest that counteracting inequitable biases requires taking bias seriously as a social and political problem and systematically attending to it in each phase—from data generation to the application of algorithms in medical practice. What the adequate approach to deal with an inequitable bias is will depend on the context in question. While we certainly do not—as Ianessi and colleagues suggest—promote this as the default solution, in some very specific cases, it could be appropriate to oversample underserved or otherwise disadvantaged populations [5, 6].

While Iannessi and colleagues agree with us that biases along social categories such as class, race, and gender exist in datasets and algorithms and that these create worse health outcomes for those patient groups against whom these algorithms are biased, they disagree with us on the point of how to deal with these biases. They argue that medicine is ‘neutral’ and should be protected from undue political influences, which they see in our suggestions to overcome inequitable biases. Iannessi and colleagues’ latter argument ignores several decades worth of research findings that show that medicine, data, and technologies are deeply social and political [7–10] and that healthcare systems often fail to treat patients equally [11, 12]—even if the people within them are genuinely committed to do so. What we show to be the case for medical data and ML in radiology is currently also visible in connection with the Covid-19 pandemic. The pandemic hit those who are marginalised and have the least economic resources the hardest. This is due to housing and working conditions, existing health status, underinsurance [13, 14], but also inequities within the medical and healthcare system itself such as implicit bias among healthcare professionals [15] or triage protocols that are based on the principle of ‘save the most lives’, which prioritize people without underlying health conditions [16]. Ignoring the social and political dimension of medicine will likely exacerbate such existing inequities and is thus certainly not in the interest of patients.

In sum, with regard to bias in radiology data and algorithms, we do not claim, of course, that datasets should be ‘diversified’ for their own sake. Correcting for biases in datasets and technologies through better representation of disadvantaged groups has the goal to increase the well-being and health status for all patients. Neither do we suggest that for the sake of equity, we should accept that algorithms are less accurate or not validated, as the authors suggest. Instead we argue that the very notion of accuracy is one that has questions about inclusion and equity already folded into it. Aiming for healthcare equity is not about creating ‘politically correct’ algorithms, and biases are not inequitable on a symbolic level because they misrepresent certain social groups. Rather, some biases are inequitable because they contribute to worse health outcomes for those people, who are already disadvantaged; this means they create and exacerbate concrete health inequities. We do, however, not advocate for creating equity through reducing the quality of healthcare that ‘privileged’ patients currently receive. In fact, it would be inequitable to withhold high-quality healthcare from anyone, if we have the means to provide it but fail to do so [17]. Countering inequitable biases means attending to bias as a social and political problem instead of merely as a technological problem that can supposedly be fixed by more data and better computer models. What to do about problems identified, however, will in each case depend on the specific practical context in which data is collected and algorithms are developed or employed.
